# Dispensing Patterns of Influenza and Pneumococcal Vaccines in the Private Healthcare Sector in South Africa for 2017 to 2021: A Longitudinal Study

**DOI:** 10.1002/pds.70477

**Published:** 2026-09-20

**Authors:** I. Truter, J. Hugo, H. Smith, S. Bansal, A. Vira

**Affiliations:** ^1^ Drug Utilisation Research Unit (DURU), Faculty of Health Sciences Nelson Mandela University Gqeberha South Africa; ^2^ Department of Pharmacy, Faculty of Health Sciences Nelson Mandela University Gqeberha South Africa; ^3^ Department of Statistics, Faculty of Science Nelson Mandela University Gqeberha South Africa; ^4^ Quantium Health South Africa Johannesburg South Africa; ^5^ Quantium Health India Hyderabad India

**Keywords:** coronavirus disease 2019 (COVID‐19), dispensing pattern, influenza vaccine, pneumococcal vaccine, private healthcare sector, profile analysis, South Africa

## Abstract

**Purpose:**

The primary aim was to conduct a longitudinal pharmacoepidemiological study on the dispensing patterns of the influenza and pneumococcal vaccines in a section of the private healthcare sector in South Africa in the presence of the COVID‐19 vaccine to document the dispensing of these two respiratory vaccines alongside COVID‐19 vaccines.

**Methods:**

A quantitative cross‐sectional pharmacoepidemiological study on health insurance data covering 5 years (2017–2021) was conducted. The study population consisted of approximately 3.8 million individuals. Profile analysis was used to compare annual dispensing patterns of both vaccines. Ethical approval for the study was obtained.

**Results:**

The annual dispensing patterns of the pneumococcal and influenza vaccines differed significantly for the period 2017–2021 (*p*‐value < 0.01), with the influenza vaccine dispensed most frequently from March to June. A similar pattern was observed for both the pre‐COVID‐19 period (2017–2019) (*p*‐value < 0.01) and the post‐COVID‐19 period (2020–2021) (*p*‐value < 0.01). At the 5% level of significance, there was a slight increase in the dispensing pattern of pneumococcal vaccines post‐COVID‐19, when compared to the dispensing pattern pre‐COVID‐19 (*p*‐value < 0.05). For the period March to June, there was, however, a significant increase in the dispensing pattern of influenza vaccines post‐COVID‐19 (*p*‐value < 0.01).

**Conclusions:**

The presence of the COVID‐19 vaccine has collectively not had a significant impact on the dispensing patterns of pneumococcal and influenza vaccines. When considering the vaccines separately, however, for the months between March and June, significantly more influenza vaccines were dispensed post‐COVID‐19.

## Key Points

- The COVID‐19 pandemic is reported to have influenced the dispensing patterns of other non‐COVID‐19 respiratory vaccines.
- The annual dispensing patterns of the pneumococcal and influenza vaccines differed significantly for the period 2017 to 2021 (*p*‐value < 0.01), with the influenza vaccine dispensed most frequently from March to June.
- Using profile analysis, the COVID‐19 vaccine has collectively not had a significant impact on the dispensing patterns of two other important vaccines during the pandemic, namely the pneumococcal and influenza vaccines.
- When considering the two vaccines separately, however, for the months between March and June, significantly more influenza vaccines were dispensed post‐COVID‐19.

## Plain Language Summary

The study investigated the dispensing patterns of the influenza and pneumococcal vaccines over a period of 5 years (2017–2021) in South Africa to potential changes brought about by the COVID‐19 pandemic. A big database covering a large portion of the private healthcare sector in South Africa was used for the study. Between groups, the annual dispensing patterns of the pneumococcal and influenza vaccines differed significantly during the study period, with the influenza vaccine dispensed most frequently from March to June. This coincides with the start of the influenza season in South Africa. Using a statistical technique called profile analysis, it was established that, overall, the COVID‐19 vaccine did not have a significant impact on the dispensing pattern of the influenza and pneumococcal vaccines when combined. However, when considering the two vaccines separately, for the months between February and July, significantly more pneumococcal vaccines were dispensed post‐COVID‐19, with the dispensing of this vaccine peaking in March. For the pre‐COVID‐19 period, however, the dispensing of this vaccine peaked in April. This study only focused on two vaccines, but studies on childhood and travel vaccine patterns are also important, especially in light of recent outbreaks of several vaccine preventable diseases.

## 1 Introduction

Vaccination remains one of the most cost‐effective health care interventions [1]. Although the coronavirus disease 2019 (COVID‐19) pandemic has put a renewed focus on the value of vaccines, it is also reported to have influenced the vaccination patterns of other non‐COVID‐19 vaccines, especially expected for the influenza and pneumococcal vaccines. These two vaccines were considered of importance, given that both vaccines provide prevention against respiratory pathogens.

In a systematic review and meta‐analysis to assess the association between seasonal influenza vaccination and pneumococcal vaccination with SARS‐CoV‐2 infection and its clinical outcomes, it was found that influenza and pneumococcal vaccination were associated with a lower risk of SARS‐CoV‐2 infection [2].

A quantitative, cross‐sectional study [3] explored changes in childhood vaccination patterns (known as the Expanded Programme on Immunisation in South Africa [EPI‐SA]) in the private healthcare sector in South Africa. It was found that the proportion of children receiving non‐COVID‐19 vaccines was higher before the pandemic (60%) than during the pandemic (55%) [3]. Furthermore, a notable 5% decline in the measles vaccination rate was reported during the children's first year of life [3]. Although not specifically linked to the previous statement, the study also mentioned that during 2023, a measles outbreak occurred in South Africa [3]. Furthermore, recent data obtained from the United Nations Children's Fund (UNICEF) indicated that increased vaccine hesitation during the COVID‐19 pandemic resulted in South Africans' trust in childhood immunisations declining by 30%, with one in five children now being under‐immunised [4]. In other parts of the world, vaccine hesitancy has also been linked to decreased vaccination coverage. In the investigation by van Rooyen and colleagues [4] chi‐squared tests were used for comparisons among distinct groups, with statistical significance ascertained through a two‐sided probability threshold of *p* < 0.05. They concluded that in the private healthcare sector in South Africa, during the COVID‐19 pandemic, vaccination trends during a child's first year of life showed a decrease in some vaccines, while an increase was observed in other vaccines [4]. There was, however, an increase in immunisation uptake observed during a child's second year of life when compared to the period before the pandemic [4]. Although no mention was specifically made about both the influenza and pneumococcal vaccines, their findings serve as a foundational assessment of vaccine prescribing trends or patterns within a subset of the South African private healthcare sector, before and during the COVID‐19 pandemic.

The first COVID‐19 case in Taiwan was reported during January 2020 [5]. Lan and colleagues mentioned that global vaccination efforts declined in more than 90% of the countries worldwide during the COVID‐19 pandemic, and that several factors may have contributed to this decline, which include disruptions in medical services or vaccination practices, school closures and lockdowns, insufficient supplies of personal protective equipment and a global lack of medical staff and healthcare providers [5]. As part of their study, they observed that globally, public attitudes towards the COVID‐19 pandemic not only influenced vaccine hesitancy towards the COVID‐19 vaccines but also towards the influenza and pneumococcal vaccines [5]. In contrast, they observed that in Taiwan, since influenza and pneumococcal vaccines were recommended for vulnerable populations to prevent severe pneumonia, there was an increased willingness among specifically women, adults without underlying disease and younger adults, to receive both the influenza and pneumococcal vaccines after the COVID‐19 outbreak [5]. Lan and colleagues [5] used *t‐*tests to compare continuous variables, while chi‐squared tests were used to compare categorical variables. For two‐tailed tests, results were considered statistically significant if the *p‐*values were < 0.05.

Pharmacoepidemiology not only quantifies the number and types of vaccines, but can also provide epidemiological data to understand disease outbreaks and implement preventative measures. There is a paucity of published studies on vaccine dispensing on large databases in South Africa. The study by Van Rooyen and co‐investigators [4] was the first of its kind on vaccine data during the pandemic in South Africa, but did not exclusively investigate the influenza and pneumococcal vaccines.

The primary aim of this study was to conduct a longitudinal pharmacoepidemiological study on the dispensing patterns of the influenza and pneumococcal vaccines in a section of the private healthcare sector in South Africa in the presence of the COVID‐19 vaccine, to document the dispensing of these two respiratory vaccines alongside COVID‐19 vaccines.

## 2 Methods

### 2.1 Setting and Study Population

The study analysed the demographic and claims records of members belonging to private healthcare sector medical aid schemes administered by one of the largest private healthcare insurance providers in South Africa. The study population consisted of approximately 3.8 million individuals from 1.8 million households in South Africa. The database, however, only represented a section of the private healthcare sector in South Africa, with the study population consisting of members who belong to a medical insurance scheme, as opposed to patients who are served by the public (government funded) healthcare sector. It has been reported that the percentage of individuals who are covered by a medical aid scheme in South Africa changed very little between 2002 and 2023, declining only slightly from 15.9% to 15.7% over the period [6]. Given the population of South Africa, it can therefore be estimated that this study covered approximately 40% of all patients in the private healthcare sector in South Africa.

### 2.2 Study Design

A descriptive retrospective cross‐sectional pharmacoepidemiological study was conducted. Data covered a 5‐year period (2017–2021). The Anatomical Therapeutic Chemical (ATC) Classification System was used to extract all records for vaccines (ATC Group J07) [7]. The Monthly Index of Medical Specialties [8] and the South African Medicines Formulary [9] were also used to further identify and classify vaccines. All patients who were dispensed one or more vaccines were identified using the Anatomical Therapeutic Chemical (ATC) Classification System [9]. All records for vaccines (ATC Group J07) were extracted for analysis. ATC group J06 (immune sera and immunoglobulins) was not included in the study. Only products that were claimed and reimbursed by the different medical insurance schemes were included in the study. The data used represented the proportion of vaccines dispensed per scheme member per month. As mentioned therefore, for both vaccines, monthly dispensing proportions were used to ensure that the time period in between measurements for both vaccines was the same. Also, using proportions which are scaleless in the profile analysis ensured that the profiles obtained for the two vaccines were equivalent, and therefore comparable. Here, the mentioned profiles obtained either represented the dispensing patterns of the two vaccines over the study period for between group comparisons or the dispensing patterns of the two respective vaccines during the pre‐ and post‐COVID‐19 periods, used for within group comparisons. Microsoft Access and Excel were used to analyse the data. Descriptive statistics were calculated and profiles were plotted.

No clinical information or diagnoses were available in the database. This was not considered as a limitation, since vaccines are administered preventatively in this case and not to treat a specific condition.

### 2.3 Data Extraction and Analysis

Snowflake Cloud Solutions was used to store all scheme data. Structured Query Language (SQL) was used to query and extract data for analysis. Microsoft Excel was then used to analyse and present data. Statistics include the total number of vaccines (quantity) and the total number of patients relating to claims submitted to the company for each gender, age and province. A limit on the total number of claims for an individual was set as 40 vaccines per year.

### 2.4 Profile Analysis

Profile analysis was used to compare annual dispensing patterns of both mentioned vaccines. According to Rencher [10], if two independent groups have the same number of plotted means or co‐ordinates, both groups follow a p‐dimensional multivariate normal distribution. If the variables in both groups commensurate within, and between groups, the profiles of both groups can be compared. For comparative purposes, results were considered statistically significant if two‐tailed *p‐*values < 0.05.

Therefore, instead of just comparing average numbers of dispensed vaccines during different periods of the year using hypothesis tests for equality of means like previous studies have used, a more appropriate testing procedure may be provided in the form of a profile analysis [10]. Profile analysis is a multivariate statistical technique that makes use of graphical representations in order to analyse the shape or profile of variables across groups. The method is well suited for analysing vaccine dispensing patterns, where shapes of summarised dispensing data can be compared over multiple periods. Using a profile analysis, it can therefore be established if dispensing patterns of different vaccines follow the same shape or profile during the same dispensing period. The results of a profile analysis can therefore indicate a similar dispensing pattern during the same period, even though the average number of vaccines dispensed may be quite different. Should the dispensing patterns of different vaccines be similar during the same period however, a profile analysis can also be used to test if the average numbers of different vaccines dispensed differ significantly or not.

According to Rencher [10], a profile analysis can be conducted as follows. Suppose that the vector variable X follow a p‐dimensional multivariate normal distribution given by $X\sim N_{p}\left(\mu ,\Sigma \right)$, where μ represents the vector of population means of the different variables in the vector variable X, and Σ, the population covariance matrix containing the variances of the individual variables as well as covariances of pairs of variables in the vector X. Suppose further that the variables in the vector X commensurate (are measured in the same units and have approximately the same variance), the means $\mu_{1},\mu_{2},\ldots ,\mu_{p}$ in the vector μ can then be compared by plotting these means as co‐ordinates. When these co‐ordinates are connected with straight lines, the plot that results, is referred to as a profile [10]. Failing to reject the null hypothesis for a test of flatness of the profile, would then indicate that no significant difference existed over the period between the means plotted as co‐ordinates of the profile. The means plotted as co‐ordinates to form the profile, are therefore, for all practical purposes, equal.

According to Rencher [10], if two independent groups have the same number of plotted means or co‐ordinates, both groups follow a p‐dimensional multivariate normal distribution as stated above, and if the variables in both groups commensurate within, and between groups, the profiles of both groups can be compared. Three hypothesis tests would then be of interest. These are a test of parallel profiles, a coincidental or same level of profiles test, and a test of flatness of both profiles. For the parallel profiles test, two groups are compared in order to determine if the line segments of the two profiles, formed between adjacent co‐ordinates, have the same slopes or gradients. If this is the case, these two line segments follow the same pattern and are said to be parallel [10]. If the null hypothesis of parallel profiles is rejected, a $\left(p-1\right)$‐dimensional discriminant function can be determined which is given by $a={\left(CS_{\mathrm{pl}}C^{T}\right)}^{-1}C\left({\bar{X}}_{1}-{\bar{X}}_{2}\right)$, where C is a matrix of contrasts, $S_{\mathrm{pl}}$ is a pooled covariance matrix, $C^{T}$ is C transposed, and ${\bar{X}}_{1}$ and ${\bar{X}}_{2}$ are the sample mean vectors of groups one and two respectively. The line segments that produce the largest absolute value in the vector a, represent the line segments that contribute the most towards the profiles not being parallel [10]. If the null hypothesis of parallel profiles is not rejected, a coincidental, or same level test, may be conducted. For this test, the profiles of the two groups are compared to see if the two profiles fall at the same level, thus indicating that the co‐ordinates plotted for the two groups, are equal. This is done by comparing the average level of profile one, with the average level of profile two [10]. A test of flatness of the two profiles can either be done simultaneously, or alternatively, also be conducted separately for both groups in a similar way as described above for one group.

For the purpose of this study, two of the mentioned three hypothesis tests would then be of interest. These are a test of parallel profiles and a test of flatness of both profiles. The latter can be done on both profiles simultaneously or separately if the null hypothesis of parallel profiles is rejected.

Microsoft Access was used to extract the data required for the analysis, while the data analysis function in Microsoft Excel, specifically the Analysis Toolpak, was used to obtain descriptive statistics necessary for the profile analysis to be conducted. Microsoft Excel built‐in functions, specifically *mmult and minverse*, necessary to perform the matrix algebra required for the profile analysis, were also used. The built‐in graphing functions in Microsoft Excel were used to plot the profiles of interest. A statistician was consulted during this phase of the study to assist with statistical analyses.

### 2.5 Ethical Considerations

Ethics approval for the use of the database to carry out this study was granted by the Research Ethics Committee (Human) (REC‐H) at Nelson Mandela University to conduct research on electronic databases (registration number: H22‐HEA‐PHA‐001).

The data for the study was obtained through an ISPOR South Africa Data Grant Award. Quantium Health is an independent company that provides data analytics and strategic consulting services to the medical insurance administrator. Data for the study was made available as part of Quantium Health's commitment to support research initiatives with broader public health significance, and does not advise its clients on the clinical treatment of its members. The data was accessed in terms of, and under, the conditions set out in a memorandum of understanding between Quantium Health, the study investigators and the university. The data was provided in a de‐identified and aggregated format and the research team had no access to information that would enable the identification of any individual. The study was also conducted in accordance with the Protection of Personal Information (POPI) Act of South Africa [11]. To safeguard privacy and confidentiality, the data provided by the medical insurance scheme underwent anonymisation. This process entailed the removal of all identifying information relating to medical schemes, beneficiaries, and service providers. All findings are presented at an aggregate level and no confidential member, healthcare provider or scheme information is disclosed.

GenAI was not used in this study.

## 3 Results

### 3.1 General Vaccine Dispensing Information

Table 1 shows the total number of vaccines claimed and the total number of vaccine patients from 2017 to 2021.

**TABLE 1:** Number of vaccines claimed and number of vaccine patients from 2017 to 2021.

Year	Total vaccines	Total vaccine patients
2017	464 766	293 464
2018	489 858	319 758
2019	512 799	339 863
2020	592 169	339 863
2021	3 831 794	339 863

### 3.2 Monthly Proportions of the Influenza and Pneumococcal Vaccines Dispensed

Basic descriptive statistics were calculated for the monthly proportions of the influenza and pneumococcal vaccines dispensed in this section of the private healthcare sector in South Africa for the period from 2017–2021 (see Table 2). Descriptive statistics were only reported for the months from February to July.

**TABLE 2:** Basic descriptive statistics of monthly proportions of influenza and pneumococcal vaccines dispensed in the private healthcare sector in South Africa for the period 2017 to 2021.

		Proportion of vaccines dispensed			
		Influenza vaccine		Pneumococcal vaccine	
		Mean	Standard deviation	Mean	Standard deviation
2017	February	1.67778E−05	2.65884E−05	0.001115556	0.000464001
	March	0.010326778	0.002381524	0.001502111	0.000568647
	April	0.019558111	0.004069037	0.001442778	0.000500446
	May	0.009070778	0.001834916	0.001414556	0.000579205
	June	0.002034778	0.000497383	0.001133	0.00046514
	July	0.000559444	0.000222099	0.000956667	0.000415685
2018	February	6.22222E−06	8.91316E−06	0.001038778	0.000485889
	March	0.015386889	0.003571959	0.001400556	0.000579689
	April	0.02007	0.003900385	0.001576778	0.000558793
	May	0.009600889	0.001840439	0.001490222	0.000570385
	June	0.002694333	0.000590975	0.001126556	0.00044522
	July	0.00075	0.00022743	0.001032778	0.000452787
2019	February	8.77778E−06	6.57225E−06	0.001068889	0.000509232
	March	0.011888222	0.002752628	0.001409444	0.000663802
	April	0.024243333	0.005071913	0.001678444	0.000662437
	May	0.011821444	0.002672692	0.001548	0.000603663
	June	0.004036333	0.001050465	0.001138111	0.000481739
	July	0.000825778	0.00032801	0.001210556	0.000534871
2020	February	1.55556E−05	9.30203E−06	0.001167444	0.000499996
	March	0.024298333	0.006568568	0.003936556	0.001728859
	April	0.032400889	0.006806565	0.002964111	0.000933148
	May	0.006566889	0.001058383	0.001970667	0.000639657
	June	0.002482889	0.00066044	0.001449222	0.000546296
	July	0.001159667	0.000479042	0.001278333	0.000540674
2021	February	6.77778E−06	7.79066E−06	0.001160889	0.000578577
	March	0.009098667	0.002869661	0.001519556	0.000630214
	April	0.032107889	0.008053656	0.001797111	0.000641364
	May	0.015715222	0.002333866	0.001450333	0.000607082
	June	0.004388222	0.000856474	0.001078778	0.000509652
	July	0.001246778	0.00039526	0.000976778	0.000517028

In South Africa, pneumococcal vaccination includes the 23‐valent polysaccharide vaccine (PPSV23) for adults at high risk and the elderly, while conjugate vaccines (PCV13/PCV10) are used for children and high‐risk adults. Recommendations are to advise adults or those with underlying medical conditions to receive a PCV13 dose, followed by a PPSV23 dose 6–12 months later. Both PCV10 and PCV13 are included in the Expanded Programme on Immunisation (EPI) and are administered to infants and young children as part of their routine immunisation schedule. The pneumococcal conjugate vaccines are the ones that showed the largest variation, as can be seen in Table 3.

**TABLE 3:** Influenza and pneumococcal vaccine dispensed over the 5 years as a percentage of the total number of vaccines dispensed.

Vaccine type	ATC code	ATC description	Year					
			2017 (*n* = 464 766)	2018 (*n* = 489 858)	2019 (*n* = 512 799)	2020 (*n* = 592 169)	2021 (*n* = 3 831 794)	Average
Influenza vaccines	J07BB02	Influenza, inactivated, split virus or surface antigen	32.94	38.13	39.70	44.45	6.51	32.35
Pneumococcal vaccines	J07AL01	Pneumococcus, purified polysaccharides antigen	1.04	1.09	1.22	1.26	0.16	0.95
	J07AL02	Pneumococcus, purified‐polysaccharide‐antigen‐conjugated	7.61	6.98	6.67	8.84	0.87	6.19

### 3.3 Average Annual Dispensing Patterns of Both Vaccines for the Period January 2017 to December 2021

**FIGURE 1:**
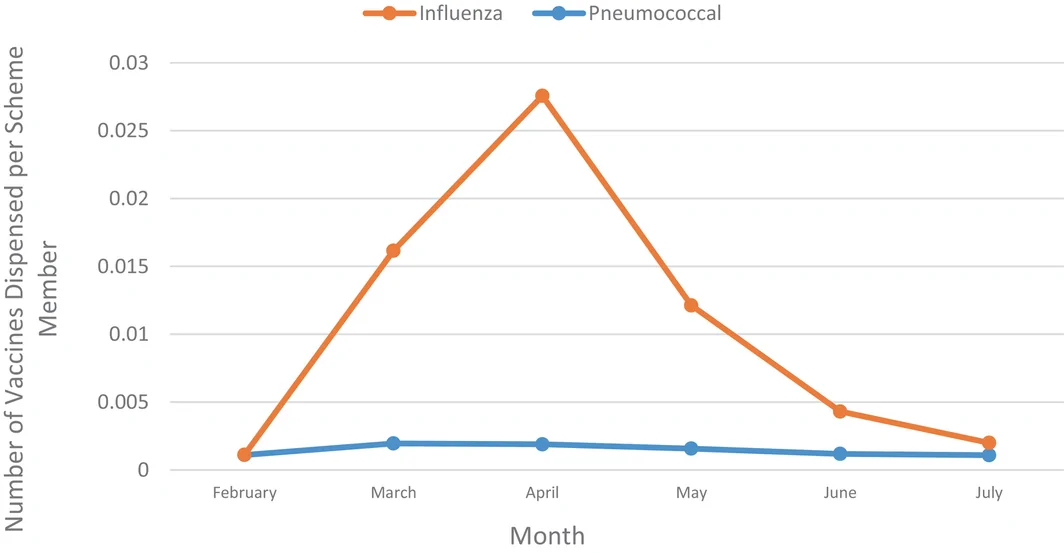
Average monthly dispensing patterns of influenza and pneumococcal vaccines for the months February–July for the period 2017 to 2021.

From the results of the profile analysis, it was evident that the annual dispensing patterns of pneumococcal and influenza vaccines differed significantly for the period 2017 to 2021 (*p*‐value < 0.01), with the influenza vaccine dispensed most frequently from March to June (see Figure 1).

**FIGURE 2:**
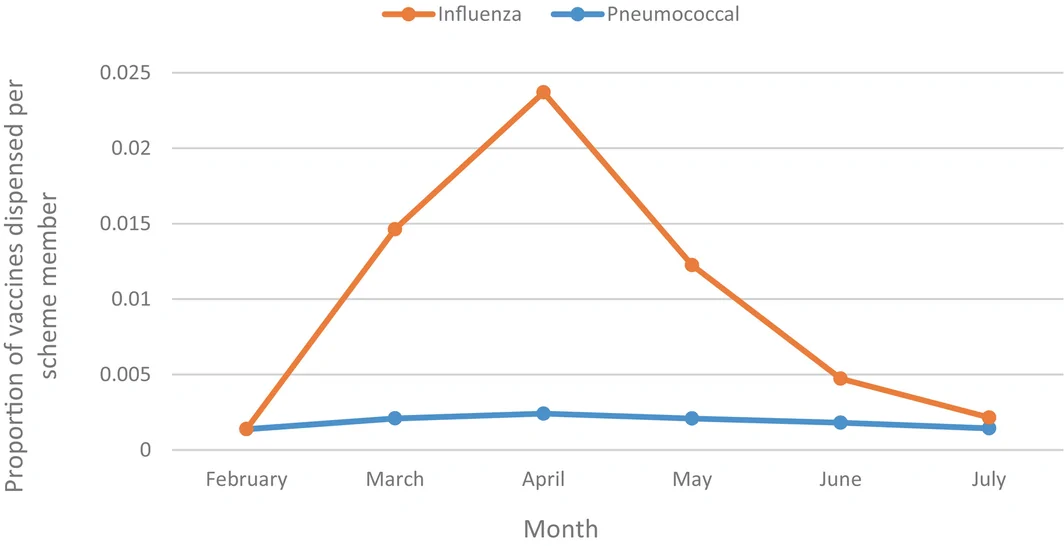
Average monthly dispensing patterns of influenza and pneumococcal vaccines for the months February–July for the period 2017 to 2019.

**FIGURE 3:**
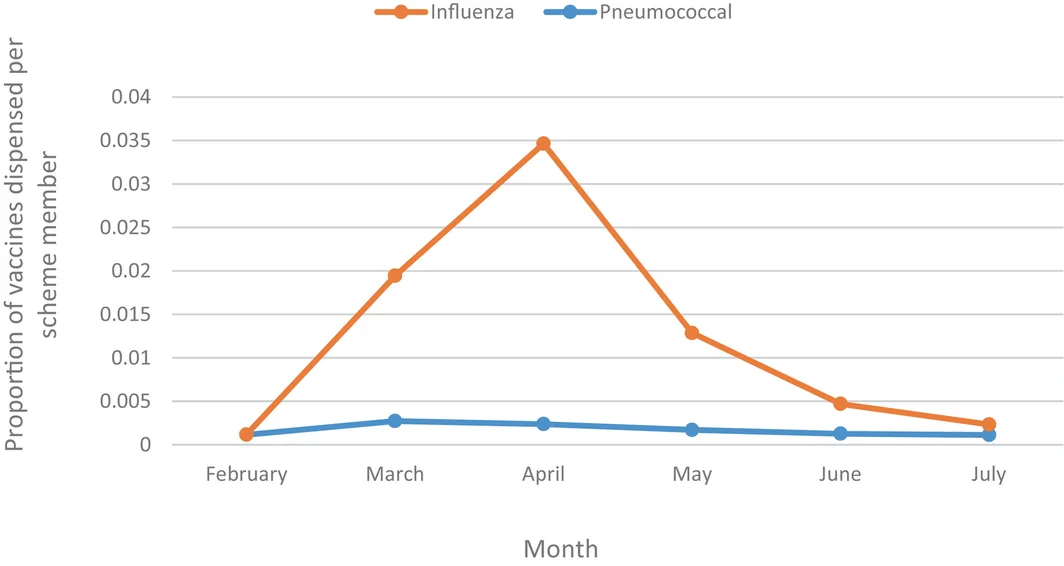
Average monthly dispensing patterns of influenza and pneumococcal vaccines for the months February–July for the period 2020 to 2021.

The same analysis was repeated, this time considering the pre‐ and post‐COVID‐19 periods separately, and only for the months from February to July (see Figures 2 and 3, respectively). From the results of the pre‐COVID‐19 profile analysis (2017–2019), it is evident that the dispensing patterns of the pneumococcal and influenza vaccines differed significantly for the months from February to July (*p*‐value < 0.01), with the influenza vaccine dispensed most frequently from March to June. The line segments between February and March and the line segments between April and May contributed the most to the profiles not being parallel. Similar results were obtained for the post‐COVID‐19 period (*p*‐value < 0.01), although an increase in the proportion of vaccines dispensed per scheme member post‐COVID‐19 was generally observed.

### 3.4 Average Monthly Dispensing Patterns of the Pneumococcal and Influenza Vaccines Considered Separately for the Pre‐ and Post‐COVID‐19 Periods

**FIGURE 4:**
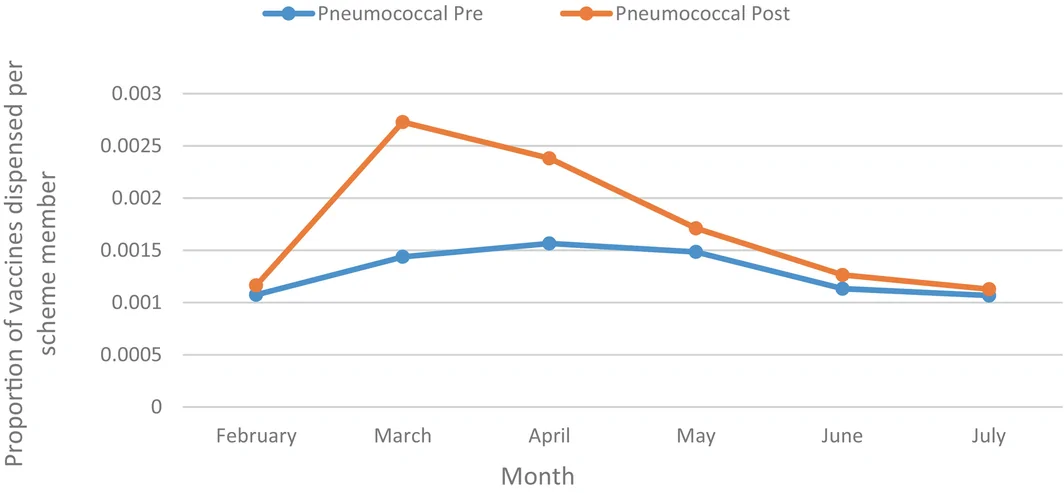
Average monthly dispensing patterns of pneumococcal vaccines pre‐ and post‐COVID‐19 for the months February to July.

When considering the pneumococcal and influenza vaccines separately, from the profile analysis it is evident that the monthly dispensing patterns of the pneumococcal vaccine differed significantly during the months from March to May between the pre‐ and post‐COVID‐19 periods (*p*‐value < 0.05) (Figure 4). Dispensing patterns were very similar, however, for February and for the period from the end of May to July between the pre‐ and post‐COVID‐19 periods. However, on average, the proportion of pneumococcal vaccines dispensed per scheme member was higher post‐COVID‐19, for the period from March to the end of May.

**FIGURE 5:**
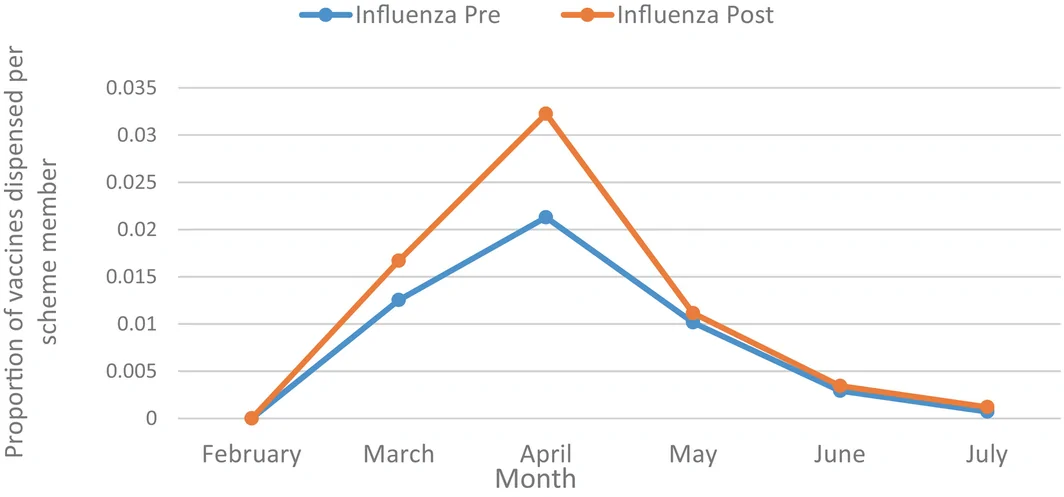
Average monthly dispensing patterns of influenza vaccines pre‐ and post‐COVID‐19 for the months February to July.

When considering the influenza vaccine, from the profile analysis it is evident that the monthly dispensing patterns of the influenza vaccine differed significantly during the months from March to May between the pre‐ and post‐COVID‐19 periods (*p*‐value < 0.01) (see Figure 5). Dispensing patterns were very similar, however, for February and for the period from the end of May to July between the pre‐ and post‐COVID‐19 periods. On average, the proportion of pneumococcal vaccines dispensed per scheme member, however, was higher post‐COVID‐19 for the period from March to the end of May. The results observed for the influenza vaccine were therefore similar to the results observed for the pneumococcal vaccine.

## 4 Discussion

The study is the largest of its kind to date in South Africa to determine the dispensing patterns of the influenza and pneumococcal vaccines. It must be recognised that the focus was on dispensing patterns from an overview perspective and not focusing on indications, diagnoses or patient‐specific demographics. It is, for example, commonly known that the 10‐valent pneumococcal conjugate vaccine is indicated for routine childhood immunisation, with dosing at 6 and 14 weeks, and then at 9 months. However, the 13‐valent conjugate vaccine is indicated in patients with immune thrombocytopenic purpura, pre‐splenectomy, as a single dose 2 weeks prior to surgery. Post‐surgery another three doses are administered. The polysaccharide vaccine is administered 8 weeks after the conjugate vaccine, and again after 5 years. These specific indications and dosage regimens were not the focus of the study. Also, one influenza vaccine claim (one vaccine per year) does not equal one pneumococcal vaccination episode, where a series of vaccines may be required. In a stabile healthcare system, the dispensing of the different vaccines in the private sector should remain relatively constant. The purpose of this manuscript was to determine if there were peaks and troughs over time in the number of vaccines dispensed, and also to determine if there were consistent patterns pre‐ and post‐COVID. We also investigated the months, including monthly deviations.

With the above background in mind, when visually considering the profile plots generated for both the influenza and pneumococcal vaccines for the period 2017 to 2021 (see Figure 1), it can be seen that the proportion of influenza vaccines dispensed in the private healthcare sector in South Africa showed a steep increase between February and April, the first 2 months of autumn in the southern hemisphere. Significantly more influenza vaccines were also dispensed when compared to the proportion of pneumococcal vaccines dispensed during the same period. The same patterns were also observed when the profiles generated for the periods between 2017 and 2019 (pre‐COVID‐19 in South Africa) and 2020 to 2021 (during and post‐COVID‐19 in South Africa) were also visually inspected (see Figures 3 and 4). As mentioned, the proportions of both vaccines dispensed per scheme member were generally higher for the period 2020 and 2021, especially during the first 2 months of autumn (March and April). These results were confirmed when the hypotheses for parallel profiles were tested for all three mentioned profile plots generated (*p*‐value < 0.01 for all three profile plots, respectively). From these results, the presence of the COVID‐19 vaccine collectively appeared not to have a significant impact on the dispensing patterns of the pneumococcal and influenza vaccines.

When considering both vaccines separately (see Figures 3 and 4), however, it is evident that the proportion of both pneumococcal and influenza vaccines dispensed per scheme member post‐COVID‐19 was higher for the period between March and the end of May, with the hypothesis tests for parallel profiles in both cases again indicating non‐parallelism (*p*‐values < 0.05 and < 0.01, respectively). What was noteworthy here was that prior to COVID‐19, a gradual increase in the proportion of dispensed pneumococcal vaccines was observed between February and April, with the proportion of this vaccine dispensed peaking in April. During and post‐COVID‐19, however, a steep increase in the proportion of dispensed pneumococcal vaccines can be observed between February and March, with the proportion of this vaccine dispensed peaking 1 month earlier in March. For the influenza vaccine, however, the same was not observed, with the proportion of this vaccine dispensed peaking in April during both the pre‐ and post‐COVID‐19 periods. Therefore, in the South African private healthcare sector, conclusions reached pertaining to influenza and pneumococcal vaccine uptake post‐COVID‐19 are therefore similar to conclusions reached in the study conducted by Lan and others [5] in the healthcare sector in Taiwan.

There are several limitations to our findings. It is important to note that the scope of this study is limited to a section of the private healthcare sector of South Africa and does not provide a comprehensive picture of the entire population of South Africa. The private sector constitutes on average about 15.7% of the South African population [6]. Similar big databases are, however, not available for the public sector. This should change with the introduction of the proposed National Health Insurance (NHI) for South Africa. The proposed NHI for South Africa includes a centralised electronic health record (EHR) system as a key component of its implementation [12]. Furthermore, this study did not include data on cash purchases of vaccines in the private healthcare sector but relied solely on medicines claims data. Despite these inherent limitations, the findings serve as a foundational assessment of vaccine prescribing trends both before and during the COVID‐19 pandemic within a large subset of South Africa's private healthcare sector.

Continuous monitoring of the vaccine landscape is needed. This study only focused on two vaccines, but studies on childhood vaccine patterns and travel vaccines are also important, especially in light of the recent outbreaks of several vaccine preventable diseases and the subsequent effects on the health of the population of South Africa.

## Author Contributions

I.T. conceptualised the study. I.T. and J.H. contributed to the study design. H.S. developed the dataset and J.H. analysed the data. S.B. and A.V. had full access to and verified the underlying study data. I.T. and J.H. drafted the manuscript. All authors contributed to the interpretation of data. All authors reviewed results, revised the manuscript, approved its final version, and agreed to be accountable for all aspects of the work.

## Funding

The authors have nothing to report. The corresponding author (Ilse Truter) was the recipient of the ISPOR South Africa Chapter and Quantium Health Data Grant for 2022 for a proposal entitled “Vaccine Dispensing in South Africa Using Electronic Health Records: A Pharmacoepidemiological Study.” This manuscript is based on the data made available through this data grant. There was no monetary value attached to the grant, and the research did not receive any other grant from funding agencies in the public, commercial or not‐for‐profit sectors. The grant merely allowed the primary researcher access to the data as requested in the bidding proposal.

## Ethics Statement

Ethical approval to conduct studies on electronic databases was obtained from the Research Ethics Committee (Human) of the Nelson Mandela University (ethics clearance number: H22‐HEA‐PHA‐001). Data for the study was made available as part of Quantium Health's commitment to support research initiatives with broader public health significance. The company does not advise its clients on the clinical treatment of its members. The data was accessed in terms of and under the conditions set out in a memorandum of understanding between Quantium Health, the study investigators and the university.

## Conflicts of Interest

I. Truter and J. Hugo have no conflicts of interest. H. Smith, S. Bansal, and A. Vira are employed by Quantium Health and assisted with data extraction, interpretation, and reporting but did not influence the findings of the study.

## Data Availability

The data that support the findings of this study are available from Quantium Health. Restrictions apply to the availability of these data, which were used under license for this study. Data are available from the author(s) with the permission of Quantium Health.
